# Endometrial Giant Cell Carcinoma Integrating Morphology, Immunophenotype, and Molecular Findings in a Misdiagnosed Case

**DOI:** 10.7759/cureus.117050

**Published:** 2026-09-28

**Authors:** Omar Y Alfaqih, Sahar B Mehecy, Mohammed Abdelatif M Elhassan

**Affiliations:** 1 Anatomical Pathology, King Fahad Medical City, Riyadh, SAU; 2 Histopathology, Sudan Medical Specialization Board, Khartoum, SDN

**Keywords:** abnormal carcinoma, carcinosarcoma mimicker, diagnostic pitfalls, endometrial cancers, endometrial giant cell carcinoma, giant cell carcinoma, giant cell tumors, immunohistochemistry, mutant p53, postmenopausal bleeding

## Abstract

Endometrial giant cell carcinoma (EGCC) is an exceptionally rare and diagnostically challenging variant of high-grade endometrial carcinoma. We describe the case of a 60-year-old postmenopausal woman who was initially misdiagnosed with carcinosarcoma on endometrial curettage. Histopathologic review, immunohistochemical analysis, and comprehensive molecular profiling identified pathogenic tumor protein p53 (TP53), phosphatidylinositol-4,5-bisphosphate 3-kinase catalytic subunit alpha (PIK3CA), and AT-rich interaction domain 1A (ARID1A) mutations, supporting the diagnosis of EGCC and its classification within the p53-abnormal (copy-number-high) molecular subtype of The Cancer Genome Atlas (TCGA). The patient subsequently underwent total abdominal hysterectomy with bilateral salpingo-oophorectomy and bilateral pelvic lymphadenectomy, which revealed no residual carcinoma and no nodal metastasis. Long-term follow-up was unavailable because the patient missed all surveillance visits, preventing any assessment of prognosis. This report underscores the importance of integrating morphologic, immunophenotypic, and molecular findings to distinguish EGCC from carcinosarcoma and other giant cell-rich uterine tumors, an approach that is essential for accurate diagnosis and for guiding individualized management of these malignancies.

## Introduction

Endometrial carcinoma represents the sixth most common malignancy among women worldwide, accounting for approximately 4.3% of all cancers in females, with an estimated 417,000 new cases and 97,000 deaths annually [1]. Despite its prevalence, the 2020 World Health Organization classification of female genital tumors has streamlined the categorization of most endometrial carcinomas into prognostically relevant types based on histopathology and molecular features; however, several rare histologic variants, including endometrial giant cell carcinoma (EGCC), remain unclassified or incompletely characterized [2].

EGCC was first described by Jones et al. in 1991 in a series of six patients [3]. Since then, only 14 further cases have been reported, giving a total of 20 published cases between 1991 and 2025 [3-11]. The present case is the 21st, and all cases are summarized in a table.

Histologically, EGCC is defined by the predominance of sheets and clusters of highly pleomorphic mononuclear and multinucleated giant cells exhibiting marked nuclear atypia, abundant eosinophilic cytoplasm, prominent nucleoli, atypical mitoses, and frequent emperipolesis [2,4,10,11]. These giant cells are often admixed with a variable component of conventional high-grade endometrioid, serous, clear cell, or undifferentiated carcinoma, which has ranged from a minor focus to as much as 85% of the tumor volume in reported cases [3,4,11]. The presence of dense lymphocytic infiltrates surrounding and infiltrating the tumor nests further contributes to its distinctive appearance [3,4].

EGCC poses significant diagnostic challenges due to its striking morphologic overlap with several aggressive uterine neoplasms, including carcinosarcoma (malignant mixed Mullerian tumor), undifferentiated/dedifferentiated endometrial carcinoma (UDEC/DDEC), and gestational trophoblastic disease [2,5,9,10].

The extreme rarity of EGCC, coupled with its alarming cytologic atypia and histologic complexity, often leads to misdiagnosis in routine pathology practice, delaying appropriate classification and management [2,5,10]. We present herein a case of EGCC in a 60-year-old postmenopausal woman, initially misinterpreted as carcinosarcoma on endometrial curettage at an outside institution. Comprehensive reevaluation incorporating detailed morphologic analysis, targeted immunohistochemistry, and next-generation sequencing enabled accurate reclassification. This report contributes to the sparse literature on EGCC, emphasizing the critical role of multimodal diagnostic approaches in resolving its mimics and refining our understanding of this enigmatic entity.

## Case presentation

A 60-year-old postmenopausal woman experienced two years of intermittent brown vaginal discharge, which changed into bright red bleeding over six months. She had no history of hormonal therapy, but her family history included a sister with uterine carcinoma.

Pelvic MRI revealed multiple small intramural and intracavitary fibroids and a 1.5 cm polypoid lesion in the lower endometrial cavity, with less than 50% myometrial invasion (Figure 1). Curettage performed at an external hospital resulted in a diagnosis of carcinosarcoma, prompting her referral.

**Figure 1:**
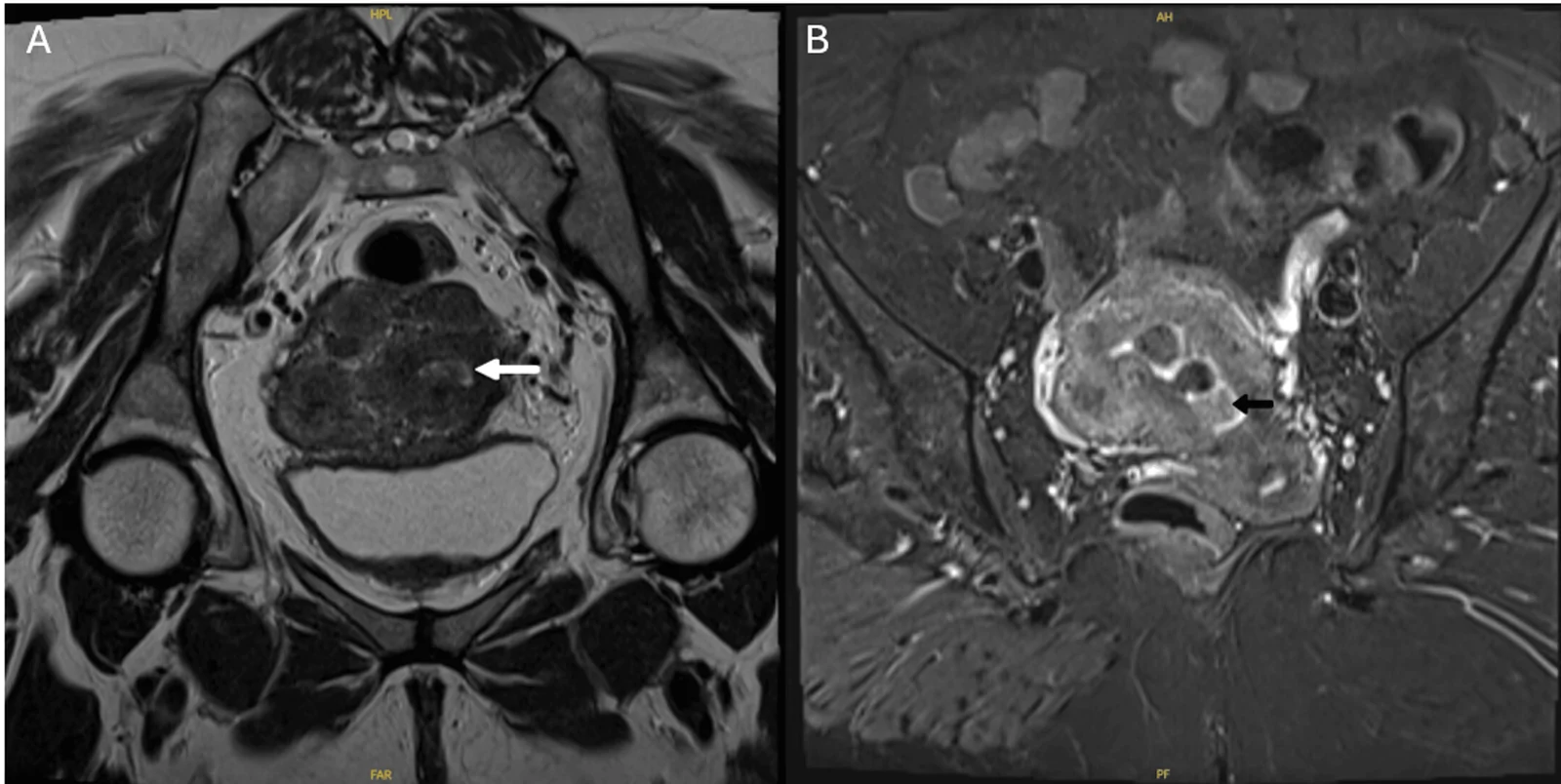
Pelvic magnetic resonance imaging (MRI): (A) axial T2-weighted image and (B) coronal post-contrast T1-weighted image demonstrating a polypoid lesion within the lower endometrial cavity. (A) The lesion shows intermediate T2 signal intensity (white arrow). (B) The lesion shows heterogeneous post-contrast enhancement (black arrow).

Microscopic examination showed a high-grade carcinoma rich in giant cells, with sheets and nests of pleomorphic multinucleated and mononuclear tumor cells. These cells had abundant eosinophilic cytoplasm, hyperchromatic nuclei, prominent nucleoli, and numerous atypical mitoses. A dense lymphocytic infiltrate surrounded and infiltrated the tumor, and emperipolesis was frequently seen. Giant cells constituted approximately 95% of the neoplasm, and approximately 5% comprised areas resembling high-grade serous carcinoma (Figure 2). No heterologous mesenchymal elements, chondroid or osteoid differentiation, or distinct sarcomatous component was identified in any of the examined sections.

**Figure 2:**
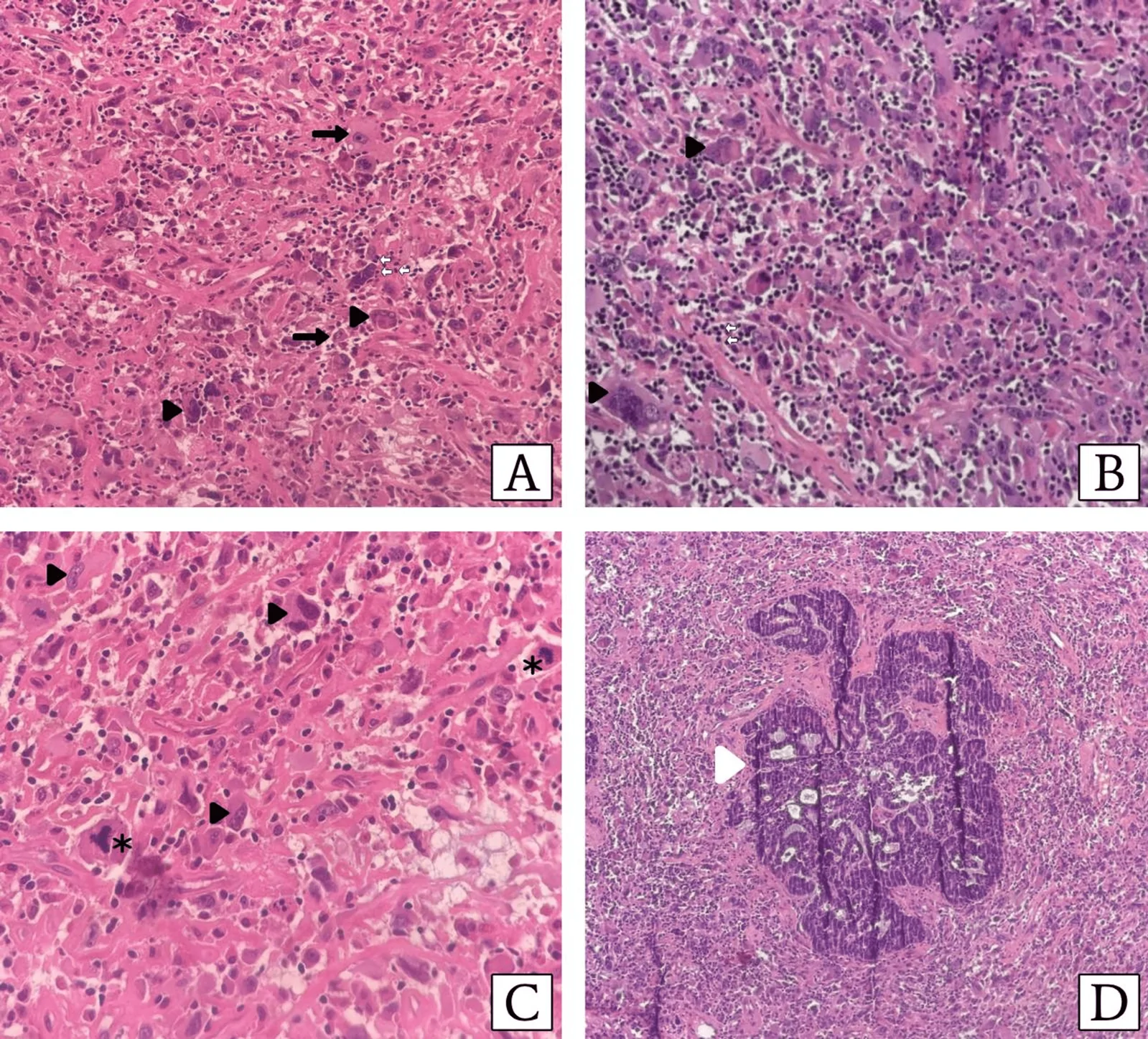
Histomorphology of endometrial giant cell carcinoma (H&E stain). (A) Low-power view showing a diffuse proliferation of pleomorphic giant (black arrowheads) and mononuclear tumor cells (black arrows) in a background of dense lymphocytic infiltration (white arrows) (H&E, ×40). (B) Intermediate-power view demonstrating marked nuclear pleomorphism, multinucleated tumor giant cells (black arrowheads), and brisk intratumoral lymphoid infiltrate (white arrows) (H&E, ×100). (C) High-power view highlighting bizarre multinucleated giant cells, abundant eosinophilic cytoplasm, prominent nucleoli, and frequent atypical mitoses (black asterisks) (H&E, ×200). (D) Focus showing a minor component of high-grade serous carcinoma adjacent to the giant cell–rich areas (white arrowhead) (H&E, ×40).

Immunohistochemistry revealed diffuse PAX8 positivity. CK7 was positive in a focal distribution, with moderate-intensity cytoplasmic staining confined to approximately 10% of tumor cells. p53 showed a mutant-type pattern of diffuse strong nuclear overexpression, and p16 was strongly and diffusely positive. Estrogen receptor showed weak patchy nuclear staining, whereas progesterone receptor was negative. Desmin, myogenin, SALL4, and p63 were negative. HER2 was scored 0, indicating negativity. All mismatch repair proteins (MLH1, PMS2, MSH2, MSH6) were retained, showing MMR proficiency (Figure 3).

**Figure 3:**
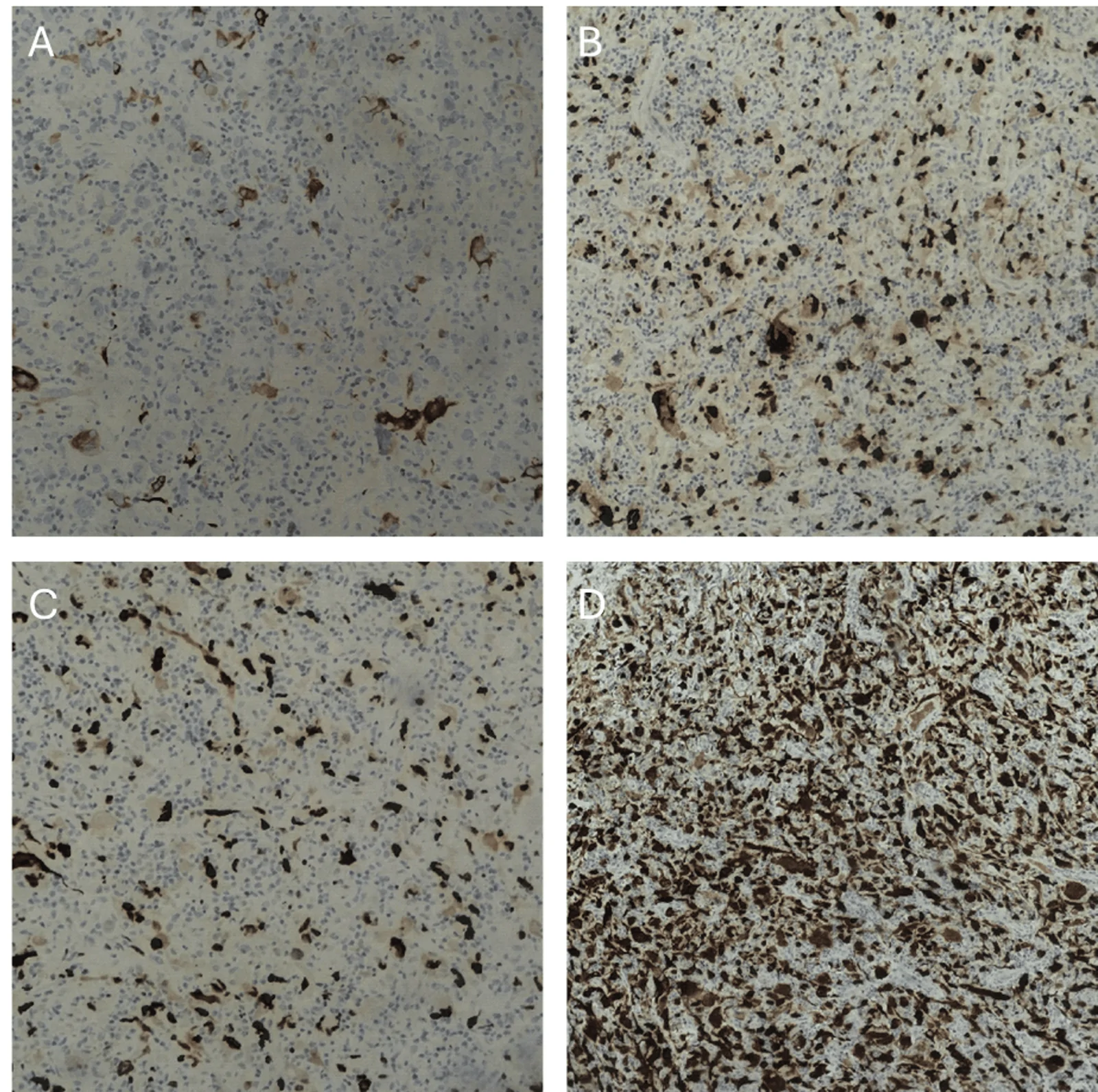
Immunohistochemical profile. (A) CK7 showing focal cytoplasmic positivity in tumor cells (×100). (B) PAX8 demonstrating diffuse nuclear positivity consistent with Müllerian epithelial differentiation (×100). (C) p53 exhibiting diffuse strong mutant-pattern nuclear overexpression (×100). (D) p16 showing diffuse strong block-type nuclear and cytoplasmic positivity (×100).

Next-generation sequencing detected pathogenic mutations in the TP53, PIK3CA, and ARID1A genes. No mutations were identified in the POLE exonuclease domain, and the tumor was not MMR-deficient. This molecular profile corresponds with previously reported EGCC cases that fall within the p53-abnormal/copy number-high pathway, rather than the POLE-mutated or hypermutated categories.

The patient subsequently underwent total abdominal hysterectomy, bilateral salpingo-oophorectomy, and bilateral pelvic lymphadenectomy. The examined specimens showed no residual carcinoma and no regional lymph node metastasis; the tumor was therefore staged as International Federation of Gynecology and Obstetrics (FIGO) stage IA on the basis of the combined curettage and hysterectomy findings. She was discharged home after surgery, having recovered well and without major events. Adjuvant therapy was not administered. The patient did not attend any of her scheduled post-operative surveillance visits and could not be contacted subsequently; consequently, no follow-up data of any duration are available for this case.

## Discussion

Endometrial giant cell carcinoma is an extremely rare and diagnostically challenging variant of high-grade endometrial carcinoma [2,5,11]. Since the original description by Jones et al. in 1991 [3], only 20 well-documented cases have been reported between 1991 and 2025; the present case therefore represents the 21st reported case and is summarised alongside the published literature in Table 1 [3-11]. The present case shows the characteristic morphology of EGCC: pleomorphic multinucleated tumour giant cells, dense lymphocytic infiltrates, emperipolesis, and a minor serous component [3,4]. The absence of heterologous elements and of desmin and myogenin expression argues strongly against carcinosarcoma [3,9].

**Table 1:** Clinicopathological and molecular features of all reported cases of endometrial giant cell carcinoma (1991-2025). Immunohistochemical and molecular data were not uniformly reported across the published cases; "not determined" indicates that molecular classification was not performed or could not be inferred from the published data. DOD: dead of disease; FIGO: International Federation of Gynecology and Obstetrics (FIGO staging is under FIGO 2009); IHC: immunohistochemistry; MMR: mismatch repair; NED: no evidence of disease; NSMP: no specific molecular profile; TCGA: The Cancer Genome Atlas; ProMisE: Proactive Molecular Risk Classifier for Endometrial Cancer

Case	Author (year)	Age (years)	FIGO stage	Giant cell component	Conventional component	IHC/biomarker status	TCGA/ProMisE subtype	Clinical outcome/follow-up
1	Jones et al. (1991) [3]	43	IA	15%	Endometrioid (85%)	Not reported	Not determined	NED at 6 years
2	Jones et al. (1991) [3]	62	IA	Predominant	Endometrioid	Not reported	Not determined	NED at 2 years
3	Jones et al. (1991) [3]	67	IA	Predominant	Endometrioid	Not reported	Not determined	Recurrence at 4 months; DOD at 3 years
4	Jones et al. (1991) [3]	69	IIA	Predominant	Endometrioid	Not reported	Not determined	DOD at 6 months
5	Jones et al. (1991) [3]	62	IVB	15%	Endometrioid (85%)	Not reported	Not determined	DOD at 5 months
6	Jones et al. (1991) [3]	85	IVB	Predominant	Clear cell	Not reported	Not determined	Alive with disease at 1 year
7	Mardi and Sharma (2008) [6]	70	IIIA	Predominant	Endometrioid	Not reported	Not determined	Not reported
8	Mulligan et al. (2010) [4]	53	IA	70%	Clear cell	MMR retained	Not determined	NED at 36 months
9	Mulligan et al. (2010) [4]	60	IB	100%	None	MMR retained	Not determined	Recurrence at 4 years with lung metastasis
10	Mulligan et al. (2010) [4]	58	IA	50%	Endometrioid with spindled areas	MMR retained	Not determined	NED at 14 years
11	Mulligan et al. (2010) [4]	83	IIIC2	30%	Endometrioid	MMR retained	Not determined	NED at 15 months
12	Mulligan et al. (2010) [4]	67	IA	90%	Serous	MMR retained	Not determined	NED at 16 months
13	Bhattacharyya et al. (2012) [7]	70	IB	80%	Endometrioid	Not reported	Not determined	Not reported
14	Sharma et al. (2016) [8]	60	IB	60%	Endometrioid	Not reported	Not determined	Not reported
15	Ayik Aydin et al. (2019) [9]	75	IIIC	Not reported	Not reported	p53 wild-type	Not determined	Not reported
16	Arafah et al. (2021) [10]	71	IA	>95%	None	MMR retained; p53 wild-type	Not determined	Not reported
17	Arciuolo et al. (2022) [5]	66	IA	Predominant	Endometrioid	MMR retained; p53 wild-type; POLE wild-type	NSMP	NED at 12 months
18	Arciuolo et al. (2022) [5]	55	IB	Predominant	Endometrioid	MMR retained; p53 wild-type; POLE wild-type	NSMP	NED at 8 months
19	Arciuolo et al. (2022) [5]	76	IB	Predominant	Serous	MMR retained; p53 mutated; POLE wild-type	p53-abnormal	NED at 3 months
20	Tang et al. (2025) [11]	55	IA	90%	Serous, with focal endometrioid	MMR retained; aberrant p53 expression	p53-abnormal	NED at 12 months, without adjuvant treatment
21	Present case	60	IA	95%	Serous (5%)	MMR retained; aberrant p53 expression; POLE wild-type	p53-abnormal	No residual carcinoma in the hysterectomy specimen; no follow-up data available

Within the TCGA framework and its clinical surrogate, ProMisE, which assigns cases sequentially by POLE status, MMR immunohistochemistry, and p53 immunohistochemistry [2], our tumour is unambiguously p53-abnormal/copy-number-high: it was POLE-wildtype, MMR-proficient, and p53-mutant by both immunohistochemistry and sequencing, and the accompanying PIK3CA and ARID1A mutations are consistent with this assignment [2,5,11]. Two molecular mimics must be excluded. POLE-ultramutated carcinomas may show bizarre giant cells and brisk lymphocytic infiltrates, yet they carry an excellent prognosis and are candidates for de-escalated adjuvant therapy [2]. MMR-deficient UDEC/DDEC typically shows MMR protein loss, SMARCA4 or ARID1A inactivation, and complete rather than patchy loss of PAX8 and cytokeratins [5]. Neither profile fits our case, in which PAX8 was diffusely positive, CK7 was focally retained, and MMR proteins were intact. Full molecular classification should therefore precede a final diagnosis of any giant cell-rich endometrial tumour [2].

Across the 21 cases summarized in Table 1, patients were aged 43-85 years, and most tumors were confined to the uterus (FIGO stages IA-IB in 15 cases). The giant cell component ranged from 15% to 100%, and the conventional component was most often endometrioid. Only five cases have been molecularly classified, all since 2022: two NSMP and three, including ours, p53-abnormal [5,11]. Outcomes have been heterogeneous, ranging from recurrence in four of six patients and three deaths within three years in the original series [3], to disease-free survival of 14 years [4].

No residual carcinoma was found in the hysterectomy specimen, most likely because the small (1.5 cm), minimally invasive polypoid lesion was removed entirely at curettage, as described previously [11]. This finding reflects the extent of disease at a single time point and carries no prognostic significance on its own. The main limitation of this report is the complete absence of follow-up: the patient did not attend post-operative surveillance, and because recurrence within four months has been documented in EGCC [3], an unrecorded recurrence cannot be excluded. Therefore, given the scant literature and outcome heterogeneity, long-term surveillance remains essential until more data elucidate the true biological behaviour of this rare variant.

## Conclusions

Endometrial giant cell carcinoma is a rare, high-grade variant of endometrial carcinoma that is easily mistaken for other giant cell-rich uterine tumors. This case shows that integrating morphology, immunohistochemistry, and full molecular classification enables accurate diagnosis and prevents misclassification. However, the absence of follow-up data precludes any conclusions about prognosis, and a single case cannot define the behaviour of this entity. Larger series of molecularly classified cases with long-term follow-up are therefore needed to establish the natural history and optimal management of EGCC. By adding a molecularly characterized case to the limited literature, this report highlights the importance of recognizing this rare entity to avoid misdiagnosis.
